# 

**Published:** 1892-05

**Authors:** 


### CONTENTS:

ORIGINAL ARTICLES: PAGE

A Consideration of Actinomycosis.. By Dr. F. S. Billings ...... 269
Warranty. By the Hon. John S. McKinley ...... 295
National and International Meat Inspection. By W. L. Williams ...... 309
Horseshoeing—A Criticism. By James Hamill, V. S ...... 315
Notes on Parasites. By C. W. Stiles, A. M. Ph. D ...... 319
Hæmorrhagic Anæmia in Cow. By W. H. Ridge, V. M. D ...... 326
Report of Cases of Cerebro Spinal Meningitis. By T. O. Hinebauch ...... 327

SOCIETY PROCEEDINGS:

New Jersey Veterinary Medical Association ...... 330
North Dakota State Veterinary Medical Association ...... 331
Western Iowa Veterinary Medical Association ...... 332

